# Unraveling the Ordered Phase of the Quintessential Hybrid Perovskite MAPbI_3_—Thermophysics to the Rescue

**DOI:** 10.1021/acs.jpclett.2c02208

**Published:** 2022-09-07

**Authors:** Pelayo Marín-Villa, Ana Arauzo, Kacper Drużbicki, Felix Fernandez-Alonso

**Affiliations:** † Materials Physics Center, CSIC-UPV/EHU, Paseo Manuel de Lardizabal 5, 20018 Donostia - San Sebastian, Spain; ‡ Instituto de Nanociencia y Materiales de Aragón (INMA), CSIC-Universidad de Zaragoza, Pedro Cerbuna 12, 50009 Zaragoza, Spain; § Centre of Molecular and Macromolecular Studies, Polish Academy of Sciences, Sienkiewicza 112, 90-363 Lodz, Poland; ∥ Donostia International Physics Center (DIPC), Paseo Manuel de Lardizabal 4, 20018 Donostia - San Sebastian, Spain; ⊥ IKERBASQUE - Basque Foundation for Science, Plaza Euskadi 5, 48009 Bilbao, Spain

## Abstract

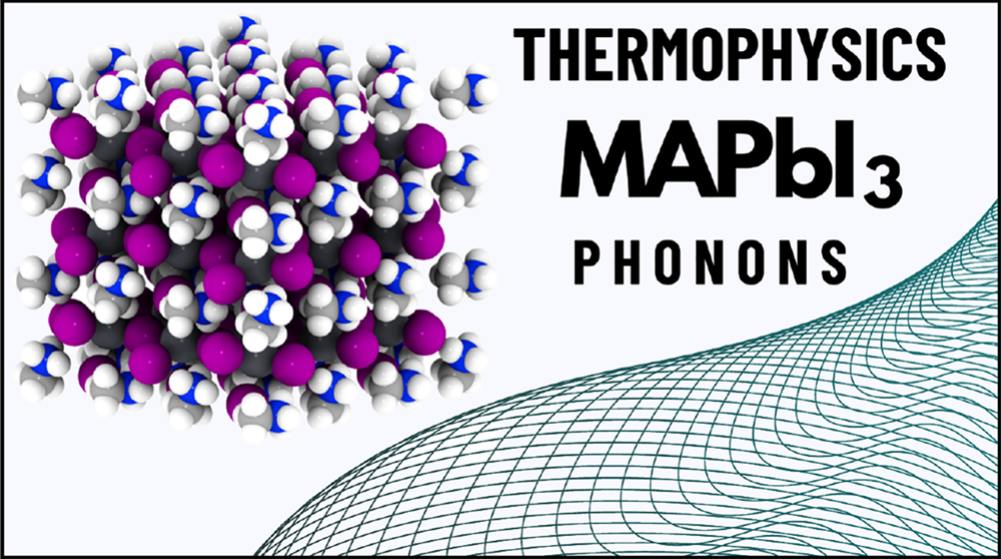

Hybrid perovskites continue to attract an enormous amount of attention, yet a robust microscopic picture of their different phases as well as the extent and nature of the disorder present remains elusive. Using specific-heat data along with high-resolution inelastic neutron scattering and ab initio modeling, we address this ongoing challenge for the case of the ordered phase of the quintessential hybrid-perovskite MAPbI_3_. At low temperatures, the specific heat of MAPbI_3_ reveals strong deviations from the Debye limit, a common feature of pure hybrid perovskites and their mixtures. Our thermophysical analysis demonstrates that the (otherwise ordered) structure around the organic moiety is characterized by a substantial lowering of the local symmetry relative to what can be inferred from crystallographic studies. The physical origin of the observed thermophysical anomalies is unequivocally linked to excitations of sub-terahertz optical phonons responsible for translational–librational distortions of the octahedral units.

In the light of the growing need for improved photovoltaic and optoelectronic materials, hybrid organic–inorganic perovskites (hereafter HOIPs) stand out as exceedingly promising candidates. They offer exceptional conversion efficiencies and coherent photocarrier transport typical of classic crystalline inorganic semiconductors,^1,2^ yet at the same time they also exhibit rather unusual nuclear dynamics reminiscent of that seen in liquids.^3,4^ While understanding their atomic structure and dynamics is critical to both rationalize their exceptional photovoltaic performance and improve their environmental and operational stability,^5^ this task remains a formidable endeavor that has to date met with limited success.

Three-dimensional HOIPs commonly exhibit a classic ABX_3_ perovskite architecture, whereby a cuboctahedral cavity is occupied by an organic cation at the A-site. These materials reveal a rich and complex phase behavior largely dictated by cation mobility,^6^ typically leading to the emergence of an ordered (orthorhombic) phase at low temperatures, followed by an intermediate tetragonal phase and a high-temperature cubic structure.^7^ However, even in the case of prototypical methylammonium lead halides like MAPbI_3_ or MAPbBr_3_, a consistent picture of their short- and long-range structures remains a challenge to both experiment and computation. This is even the case well within the ordered phase at low temperatures, where the effects of dynamical disorder associated with either the organic cation or the surrounding inorganic framework are minimized.^8−13^ Moreover, the A-site organic molecules and corner-linked PbX_6_ octahedra exhibit strong dynamical couplings giving rise to preferred short-range ordering of the organic cations, accompanied by distortions of the perovskite framework in the vicinity of the guest molecules. It is common for these local distortions to be observed by probes of local structure rather than by crystallographic techniques sensitive to the long-range order.^6,14^ In addition to the above, a robust structural characterization of HOIPs is further hampered by their high propensity to exhibit crystal twinning^15,16^ and nanoscale domains of different origins,^14,17−19^ once more calling for the use of probes sensitive to local structure. On the experimental front, NMR and radiation-scattering techniques (photon, neutron) can be deployed in a complementary manner to obtain much-needed input into the above, focusing on either the organic or inorganic constituents.^20^ At the same time, modern computational methodologies, particularly those relying on first-principles, have become increasingly successful at predicting the local atomic environment and its associated spectroscopic response, thereby enabling the development of models increasingly closer to experimental observation. In our previous works, such a combined experimental and computational strategy was applied for the first time to the case of the ordered phase of MAPbI_3_.^21^ High-resolution inelastic neutron scattering (INS) along with first-principles modeling served to provide substantial evidence for a reduction of the local symmetry around the organic cation relative to the average *Pnma* crystallographic structure. In this case, the disruption of a fully fledged hydrogen-bonding network is accompanied by short-range octahedral-tilting distortions around the cations. In subsequent studies,^22,23^ this improved model (hereafter denoted *P*1) was used with success in the study of both MA^+^ and formamidinium (FA^+^) cations in more complex MA_1–*x*_FA_*x*_PbI_3_ solid solutions. In particular, it was possible to quantify the extent of disorder as well as to shed new light onto the mechanism of physical stabilization of the perovskite framework in these materials. Building upon the above, this work introduces the use of specific heat data to scrutinize further the validity of these two models of MAPbI_3_. Using these thermophysical data alongside phonon calculations obtained with plane-wave density functional theory (PW-DFT), we introduce a joint experimental–computational protocol allowing for a quantitative validation of candidate structural models of HOIPs. Through its connection to the Vibrational Density of States (VDoS), our quantitative specific heat predictions are further used as a stringent test of the performance of modern density functional approximations (DFAs) in terms of their cost and accuracy to describe MAPbI_3_.

To set the scene, Figure 1 provides a summary of the current state of affairs concerning the ordered structure of MAPbI_3_, based on new computational predictions using harmonic lattice dynamics (HLD) calculations and extensive benchmarking with several DFAs: PBE-TS,^24^ PBE-D3(BJ),^25,26^ PBEsol,^27^ and rSCAN.^28,29^ These calculations capitalize from our previous works^21,22^ and those of Bokdam et al.^30,31^ As shown by the INS data in Figure 1a, sharp spectral features reflect a well-defined local structure, with no discernible inhomogeneous broadenings that would otherwise signal the emergence of static disorder or the presence of distinct domains. Below 20 meV, these features are associated with librational modes of MA^+^, and they reflect a tendency for it to arrange itself in preferential directions. These observations are not compatible with the formation of an orientational glass.^22,23^ Similar considerations apply to the sharp feature observed at ca. 37 meV, associated with a disrotatory internal mode of the cation, This particular mode is a very sensitive probe of the geometry of the (rather weak) N–H···I hydrogen bonds.^22^ As extensively discussed in ref (22), the *Pnma* model cannot reproduce quantitatively a number of INS features, particularly those associated with librational motions of the organic cation. Absolute deviations from the INS data shown in Figure 1a amount to as much as 10 meV. Furthermore, these results depend far more weakly on the DFA employed, as highlighted in Figure 1b. In particular, the absence of a distinct trend in predicted transition energies between van der Waals (vdW) corrected and pure semilocal DFAs indicates that this type of interaction does not have a substantial impact on the vibrational properties of MAPbI_3_. This finding is in line with the results obtained by Pérez-Osorio et al.,^32^ which further indicates that vibrational transition energies in the ordered phase of MAPbI_3_ are not strongly dependent on other factors such as anharmonicity or spin–orbit coupling. These considerations are at odds with those Zhang et al., suggesting that vdW interactions between the organic cation and its neighboring inorganic sublattice contribute to the total electronic energy between these two components by as much as 13.4%.^33^ However, we note that this latter work was limited to the use of a highly specific, nonlocal vdW-DF2 functional, hardly benchmarked for hybrid perovskites. As such, further verification is in order. In the *Pnma* structure, MA^+^ electric dipoles are arranged in a head-to-tail fashion so as to maximize the number of hydrogen-bonding interactions with the surrounding iodine atoms. The observed overestimation in predicted transition energies relative to experiment thus indicates overbinding of MAPbI_3_ to the negatively charged inorganic framework.^23^ A substantial reduction in the local symmetry around the organic cation leading to the *P*1 model becomes necessary to remove these discrepancies.^22^ This structural model provides a superb description of vibrational transition energies, with absolute deviations below 2 meV (cf. the right panels in Figure 1b). The phonon band structures shown in Figure S1 provide additional insights into the performance of different DFAs, particularly at low energies. These data confirm that both structural models are mechanically stable at 0 K because no imaginary modes are observed. Interestingly, we find a high degree of consistency in the predicted phonon band structures for all DFAs considered. In particular, the phonon dispersion relations obtained with PBEsol are virtually identical with those obtained with the more elaborate PBE-D3(BJ) functional.^22^ Furthermore, we find a similar performance of the celebrated meta-GGA SCAN functional in its numerically regularized incarnation (rSCAN), yet at a considerably higher cost by ca. 1 order of magnitude. In line with the more recent work of Lahnsteiner et al.,^31^ we conclude that PBEsol emerges as the most versatile DFA: it is sufficiently cost-effective to describe phonon properties and finite-temperature cation dynamics with reasonable accuracy, and it represents a good alternative relative to the more demanding SCAN approach. On the basis of these results, the remainder of this work will focus on PBEsol outputs.

**Figure 1:**
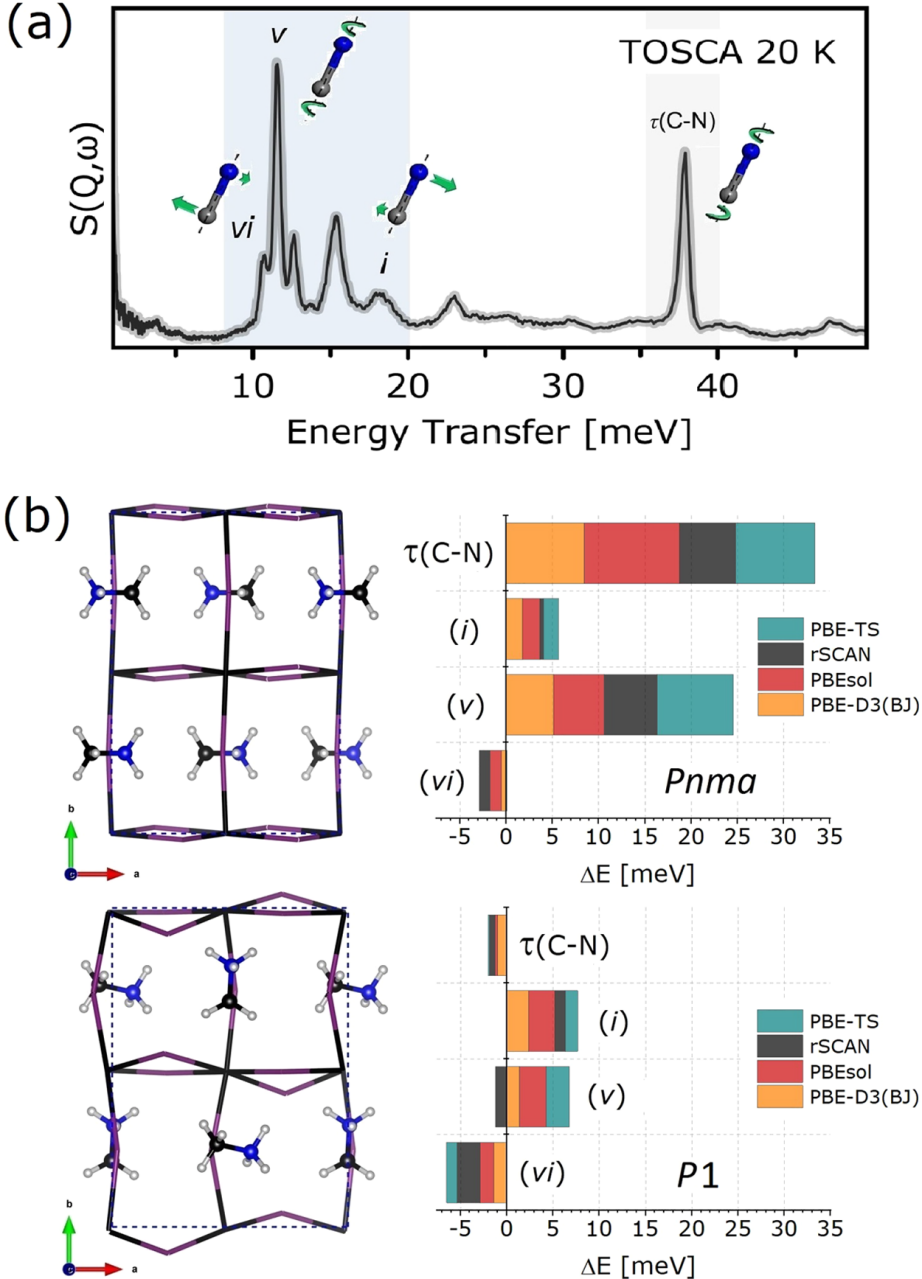
(a) INS spectrum of MAPbI_3_^22^ along with characteristic modes depicted as colored insets. The lowest-lying molecular internal (disrotatory) mode is marked as τ(C–N). The highest- and lowest-energy molecular librations are marked as (*i*) and (*vi*), respectively. The most intense librational feature marked as (*v*) arises from the reorientation of the whole MA^+^ about the C–N axis. (b) Fully optimized DFT structures of MAPbI_3_ (left panels) according to *Pnma* (middle) and *P*1 (bottom) models. The two plots on the right show the corresponding errors in predicted transition energies for modes τ(C–N), (*i*), (*v*), and (*vi*), using several DFAs (see main text for further details).

Figures 2a and 2b show the phonon band structure for the structural models considered in this work. These predictions are also compared to the INS data of MAPbI_3_ reported by Ferreira and coauthors.^4^ Given the large incoherent scattering from hydrogen, the INS data are primarily sensitive to the position of vibrational features associated with hydrogen motions in MAPbI_3_,^34,35^ thereby limiting to some extent a direct comparison with the phonon band structure obtained in the calculations. Nonetheless, at a semiquantitative level, the lowering of symmetry in going from *Pnma* to *P*1 leads to an increase in spectral congestion between 2.5 and 4 meV which is more in accord with the experimental data. In this context, we note that the possibility of using perdeuterated specimens to circumvent this limitation is not a viable option, given the preponderance of isotope effects in this material.^36^ Our calculated phonon band structures grant us, nonetheless, with the opportunity to provide quantitative predictions for the temperature dependence of the heat capacity within the harmonic approximation and to compare these with the experimental data (see also the Supporting Information). Specific predictions for the *Pnma* and *P*1 models using PBEsol are shown and compared with experimental data in Figure 2c,d over the range 2–75 K. Because the computed heat capacities have been obtained from the cumulative contributions of all normal modes, it is important to note that the present comparison is therefore performed in absolute terms, without any ad-hoc normalization of computed thermophysical data. In line with previous works,^37^ we make use of a “Debye-reduced” representation of the form *C*_*p*_(*T*)/*T*^3^ to highlight departures from Debye’s law. Additional calculations using a wider set of DFAs are further presented in Figure S2, yet we anticipate that our conclusions as explained below are not dependent on the specific DFA used to obtain the phonon band structure. The experimental specific heat data shown in Figure 2 are quantitatively in line with the results of Fabini et al.,^38^ exhibiting a broad peak centered at 5.9 K in *C*_*p*_(*T*)/*T*^3^. Further inspection of these data also shows that this low-temperature feature can be clearly reproduced by our HLD calculations, without any assumption on glass formation or disorder. It is, instead, an intrinsic feature of a fully ordered structure, arising from the presence of a dense manifold of normal modes in the VDoS in the millielectronvolts range. The *P*1 model also provides a better description of the experimental data both above and below the maximum in *C*_*p*_(*T*)/*T*^3^ observed in the laboratory. Qualitatively, this closer agreement arises from a broader distribution of low-energy phonon features in the VDoS, which is also visible in the neutron data in Figure 2a,b as a result of the lower symmetry of *P*1 relative to *Pnma*. These considerations are shared by all the DFAs tested in this work, as reported in Figure S2.

**Figure 2:**
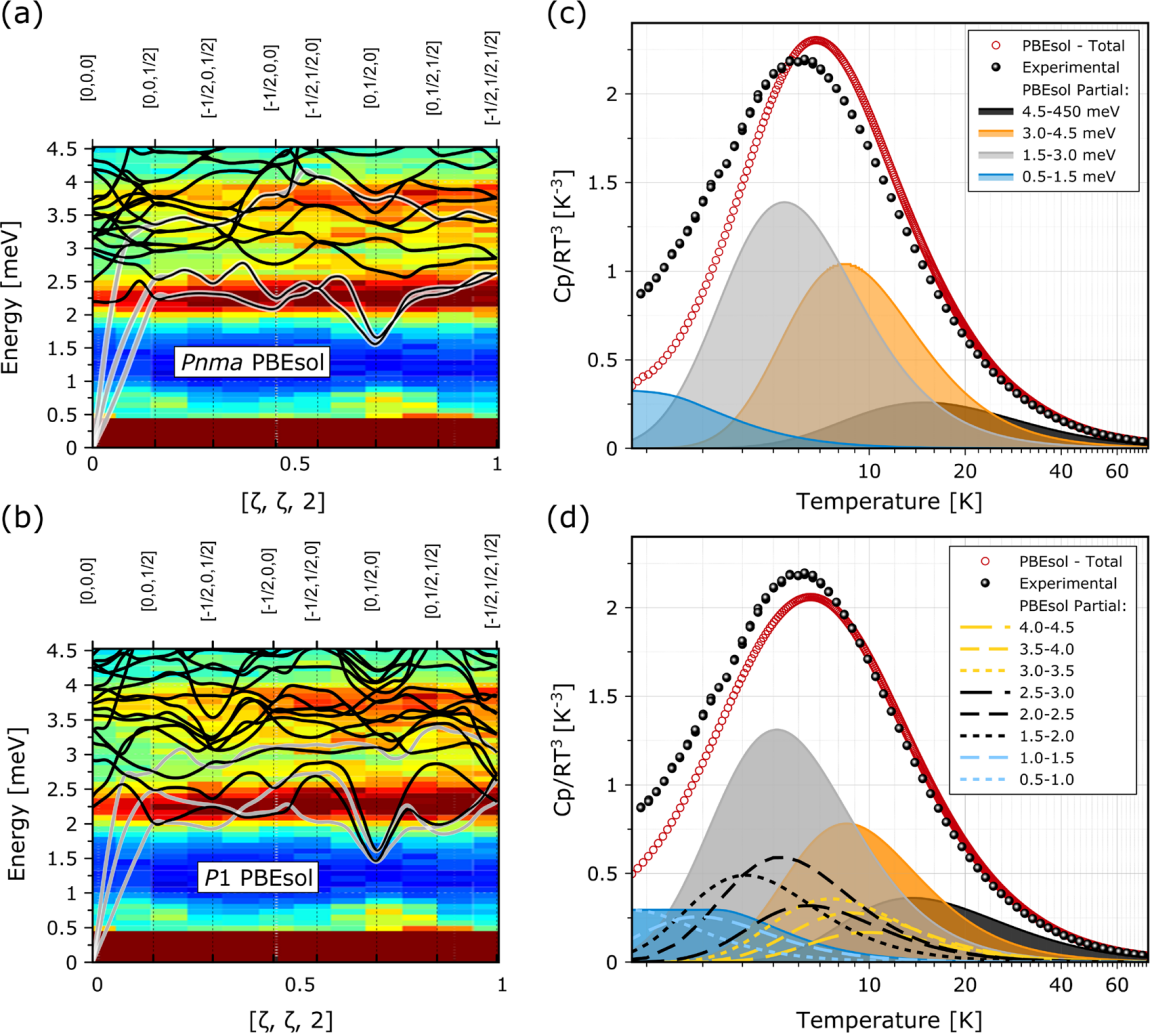
(left panels) Calculated phonon dispersion relations for the *Pnma* (a) and *P*1 (b) models. The resulting phonon branches are presented in the range 0–4.5 meV and overlaid on the color maps corresponding to INS data from a hydrogenous single-crystal specimen.^4^ (right panels) Experimental (black dots) and theoretical (red dots) *C*_*p*_(*T*)/*T*^3^. Partial contributions to this quantity from different phonon energy intervals are represented by the shaded curves, as defined in panel c. For the *P*1 model, these contributions are decomposed further by using 0.5 meV intervals, as shown by the broken lines in (d).

To scrutinize the different phonon contributions further, we have decomposed the predicted *C*_*p*_(*T*)/*T*^3^ into partial contributions over selected energy intervals (see Figure 2c,d). With the benefit of hindsight, we note that the thermophysical data exhibit a good and (largely unforeseen) degree of “resolving power” in the energy domain, via its mapping onto the temperature axis. This dissection exercise also tells us that the dominant contributions come from excitations of phonons at energies of 1.5–4.5 meV, i.e., predominantly optical lattice modes. The INS data in this regime (see Figures 2 and S1) also show an increase in the underlying VDoS at around 2, 3, and 4 meV, and these modes are characterized by a considerable projection onto the hydrogen atoms, reflecting a strong coupling between the cation and the inorganic framework. The Γ-point optical modes involving the highest contributions to the INS intensity are shown in Figure 3. Interestingly, these modes are linked to distortions of the *P*1 structure ultimately leading to *Pnma*. The lowest-energy contributions at around 2 meV can be ascribed to octahedral distortions driven by Pb displacements, which affect the mirror-plane symmetry. The contributions at around 3 and 4 meV are driven by I atom dynamics, reflecting librations and shearing of the PbI_6_ octahedra, respectively. This quantitative analysis provides further and rather unequivocal evidence on the specific physical origin of the strong departure from Debye’s law, which is related to the excitation of the lowest-energy optical phonons. This picture does not require the presumption of any static disorder, and therefore we anticipate that it will be of importance and relevance in the context of identifying its possible emergence in HOIPs with more complex cation compositions.

**Figure 3:**
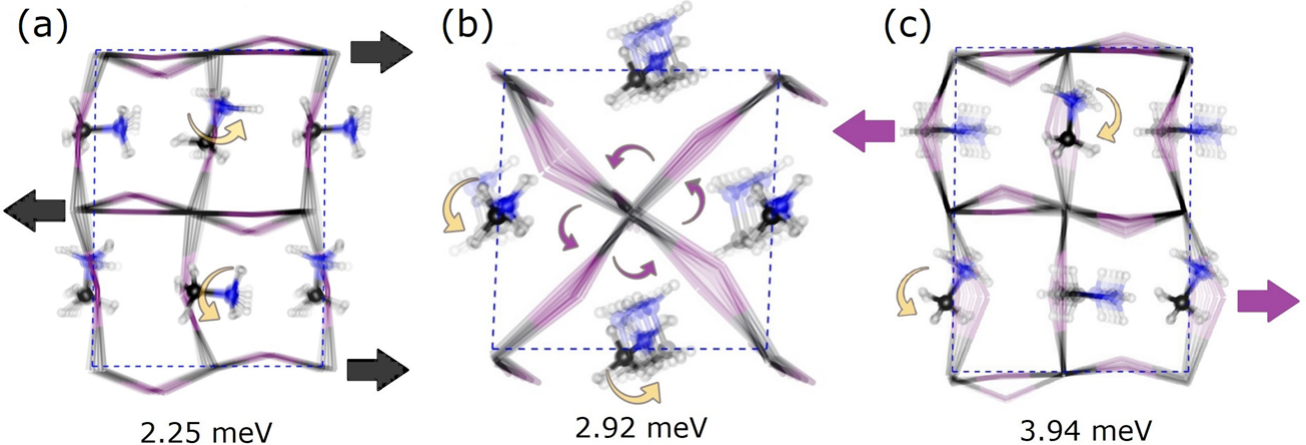
Representative Γ-point optical modes of MAPbI_3_ according to HLD PBEsol calculations using the *P*1 model. These modes correspond to the most prominent contributions from the hydrogen atoms. Modes (a) and (b) are the first and the third lowest-energy optical modes in the range 2–3 meV, associated with octahedral shearing. Mode (c) is a representative octahedral distortion contributing to the dispersion branch observed at ca. 4 meV.

As reported in Figure 4, our results can also be used to provide a quantitative assessment of the sensitivity of the heat capacity as an experimental observable to discriminate among plausible structural models as well as different DFAs. As defined in the Supporting Information, we have performed this exercise by looking at the differences between experiment and computational predictions both in terms of their temperature dependence and their associated (cumulative) mean absolute errors (MAEs) (see Figures 4a and 4b, respectively). Figure 4a displays the temperature dependence of these deviations. For *Pnma*, these are as high as 0.6–0.7 *K*^–3^ and are least pronounced for PBE-D3(BJ). PBEsol represents an interesting case with positive (negative) deviations of a similar overall magnitude below (above) the peak. This behavior indicates an overall and distinct shift of the computational predictions relative to observation. This shift is primarily caused by an overestimate of the number of normal modes in the range 3–4.5 meV. For the case of *P*1, these deviations are reduced significantly over the entire temperature range and are predominantly positive. For PBE-D3(BJ), they are below 0.2–0.3 *K*^–3^, about a 10% underestimate of the experimental values around the maximum. These considerations are further reflected in the MAEs shown in Figure 4b. The *Pnma* model performs consistently worse than *P*1 for all DFAs considered, by a factor of at least 1.5 in terms of the MAE. For the particular case of MAPbI_3_, the consistency of this result across all DFAs also tells us that these have reached a sufficiently high level of accuracy, thereby enabling a meaningful comparison between competing structural models.

**Figure 4:**
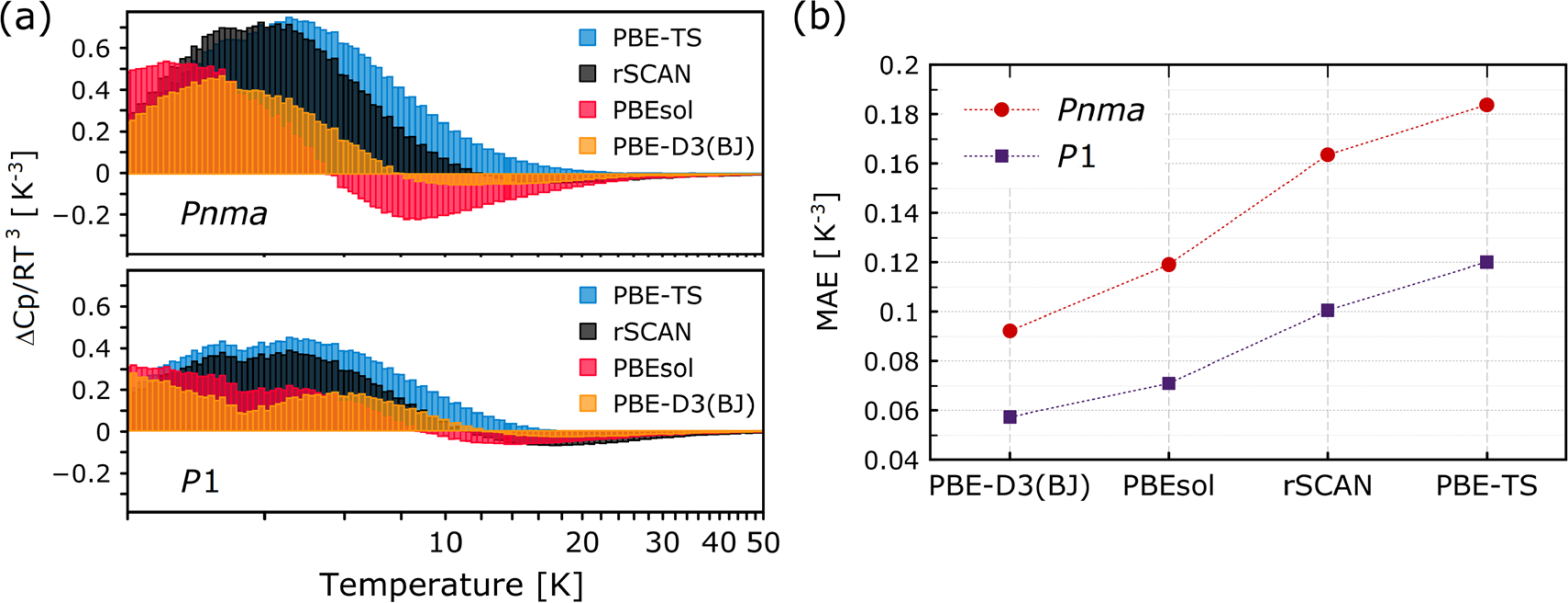
Quantitative comparison between predictions for the *Pnma* and *P*1 models. (a) Deviations from experiment using the DFAs indicated in the figure legends. For further details, see the main text. (b) Mean absolute errors (MAEs) in the predicted *C*_*p*_(*T*)/*T*^3^ using different DFAs. For details on the mathematical definitions of these deviations and MAEs, see the Supporting Information.

In summary, we have shown that a detailed scrutiny of heat capacity data constitutes a powerful approach to unravel pending questions concerning the properties of this important material. Our results also serve to demonstrate that a pseudo-orthorhombic *P*1 structure previously proposed on the basis of INS data^21^ continues to provide an improved description of the structure of the ordered phase of MAPbI_3_ at ambient pressure, relative to what has been inferred from crystallographic studies to date. We underline that this gratifying conclusion has been reached in an entirely independent manner relative to previous studies by focusing on how low-energy modes primarily associated with the inorganic framework (and not the organic cation) manifest themselves in the low-temperature behavior of a hitherto unexploited experimental observable—the heat capacity. The above program of work has been certainly facilitated by the level of quality of existing DFAs, making it possible to survey their relative performance and to ascertain that their potential limitations are now sufficiently minor to enable robust and quantitative model selection. To the best of our knowledge, this is the first time that such model selection protocol has been implemented with success in MAPbI_3_ or related materials. We also anticipate that its realm of applicability could be further extended to the study of other more complex HOIPs and their mixtures,^39−41^ where other experimental probes might also fall quite short at providing the requisite level of physical insight.

*Experimental and Computational Details*. Specific heat measurements were performed across the low-temperature phase of MAPbI_3_ down to 2 K by using a Quantum Design PPMS system. The measurements were performed on a pressed powder pellet fixed with Apiezon-N grease by using the same hydrogenous MAPbI_3_ sample as characterized elsewhere.^21^ The calculations of phonon dispersion relations were performed with CASTEP^42^ by using the finite-displacement method combined with a nondiagonal supercell approach to solve for the dynamical matrix.^43^ Further computational details are provided in the Supporting Information.

## Supporting Information Available

Computational details; deviations and statistical methods; benchmark calculations of phonon band structure; benchmark calculations of heat capacity (PDF)

## References

[ref1] Gonzalez-Pedro V.; Juarez-Perez E. J.; Arsyad W.-S.; Barea E. M.; Fabregat-Santiago F.; Mora-Sero I.; Bisquert J. General Working Principles of CH_3_NH_3_PbX_3_ Perovskite Solar Cells. Nano Lett. 2014, 14, 888–893. 10.1021/nl404252e. 24397375

[ref2] Ferreira A. C.; Létoublon A.; Paofai S.; Raymond S.; Ecolivet C.; Rufflé B.; Cordier S.; Katan C.; Saidaminov M. I.; Zhumekenov A. A.; et al. Elastic Softness of Hybrid Lead Halide Perovskites. Phys. Rev. Lett. 2018, 121, 1–12. 10.1103/PhysRevLett.121.085502. 30192590

[ref3] Miyata K.; Atallah T. L.; Zhu X. Y. Lead Halide Perovskites: Crystal-liquid Duality, Phonon Glass Electron Crystals, and Large Polaron Formation. Sci. Adv. 2017, 3, 1–11. 10.1126/sciadv.1701469. PMC5640380 29043296

[ref4] Ferreira A. C.; Paofai S.; Letoublon A.; Ollivier J.; Raymond S.; Hehlen B.; Ruffle B.; Cordier S.; Katan C.; Even J.; Bourges P. Direct Evidence of Weakly Dispersed and Strongly Anharmonic Optical Phonons in Hybrid Perovskites. Commun. Phys. 2020, 3, 1–10. 10.1038/s42005-020-0313-7.

[ref5] Mozur E. M.; Neilson J. R. Cation Dynamics in Hybrid Halide Perovskites. Annu. Rev. Mater. Sci. 2021, 51, 269–291. 10.1146/annurev-matsci-080819-012808.

[ref6] Frost J. M.; Walsh A. What Is Moving in Hybrid Halide Perovskite Solar Cells?. Acc. Chem. Res. 2016, 49, 528–535. 10.1021/acs.accounts.5b00431. 26859250 PMC4794704

[ref7] Lee J.-W.; Seo S.; Nandi P.; Jung H. S.; Park N.-G.; Shin H. Dynamic Structural Property of Organic-inorganic Metal Halide Perovskite. iScience 2021, 24, 101959–14. 10.1016/j.isci.2020.101959. 33437939 PMC7788097

[ref8] Whitfield P. S.; Herron N.; Guise W. E.; Page K.; Cheng Y. Q.; Milas I.; Crawford M. K. Structures, Phase Transitions and Tricritical Behavior of the Hybrid Perovskite Methyl Ammonium Lead Iodide. Sci. Rep. 2016, 6, 35685–16. 10.1038/srep35685. 27767049 PMC5073364

[ref9] Rakita Y.; Bar-Elli O.; Meirzadeh E.; Kaslasi H.; Peleg Y.; Hodes G.; Lubomirsky I.; Oron D.; Ehre D.; Cahen D. Tetragonal CH_3_NH_3_PbI_3_ is Ferroelectric. Proc. Natl. Acad. Sci. U. S. A. 2017, 114, E5504–E5512. 10.1073/pnas.1702429114. 28588141 PMC5514731

[ref10] Ren Y.; Oswald I. W. H.; Wang X.; McCandless G. T.; Chan J. Y. Orientation of Organic Cations in Hybrid Inorganic-Organic Perovskite CH_3_NH_3_PbI_3_ from Subatomic Resolution Single Crystal Neutron Diffraction Structural Studies. Cryst. Growth. Des. 2016, 16, 2945–2951. 10.1021/acs.cgd.6b00297.

[ref11] Bari M.; Bokov A. A.; Ye Z.-G. Ferroelastic Domains and in Organic-inorganic Hybrid Perovskite CH_3_NH_3_PbBr_3_. J. Mater. Chem. C 2021, 9, 3096–3107. 10.1039/D0TC05618A.

[ref12] Breternitz J.; Lehmann F.; Barnett S. A.; Nowell H.; Schorr S. Role of the Iodide–Methylammonium Interaction in the Ferroelectricity of CH_3_NH_3_PbI_3_. Angew. Chem., Int. Ed. 2020, 59, 424–428. 10.1002/anie.201910599. PMC6972664 31609507

[ref13] Wiedemann D.; Breternitz J.; Paley D. W.; Schorr S. Hybrid Perovskite at Full Tilt: Structure and Symmetry Relations of the Incommensurately Modulated Phase of Methylammonium Lead Bromide, MAPbBr_3_. J. Phys. Chem. Lett. 2021, 12, 2358–2362. 10.1021/acs.jpclett.0c03722. 33666079

[ref14] Beecher A. N.; Semonin O. E.; Skelton J. M.; Frost J. M.; Terban M. W.; Zhai H.; Alatas A.; Owen J. S.; Walsh A.; Billinge S. J. L. Direct Observation of Dynamic Symmetry Breaking above Room Temperature in Methylammonium Lead Iodide Perovskite. ACS Energy Lett. 2016, 1, 880–887. 10.1021/acsenergylett.6b00381.

[ref15] Szafrański M.; Katrusiak A. Photovoltaic Hybrid Perovskites under Pressure. J. Phys. Chem. Lett. 2017, 8, 2496–2506. 10.1021/acs.jpclett.7b00520. 28498672

[ref16] Breternitz J.; Tovar M.; Schorr S. Twinning in MAPbI_3_ at Room Temperature Uncovered Through Laue Neutron Diffraction. Sci. Rep. 2020, 10, 16613–8. 10.1038/s41598-020-73487-1. 33024187 PMC7538425

[ref17] Evans P. E.; Pink M.; Zhumekenov A. A.; Hao G.; Losovyj Y.; Bakr O. M.; Dowben P. A.; Yost A. J. Rotationally Free and Rigid Sublattices of the Single Crystal Perovskite CH_3_NH_3_PbBr_3_ (001): The Case of the Lattice Polar Liquid. J. Phys. Chem. C 2018, 122, 25506–25514. 10.1021/acs.jpcc.8b08994.

[ref18] Leonhard T.; Röhm H.; Altermann F. J.; Hoffmann M. J.; Colsmann A. Evolution of Ferroelectric Domains in Methylammonium Lead Iodide and Correlation with the Performance of Perovskite Solar Cells. J. Mater. Chem. A 2021, 9, 21845–21858. 10.1039/D1TA06290E.

[ref19] Vorpahl S. M.; Giridharagopal R.; Eperon G. E.; Hermes I. M.; Weber S. A. L.; Ginger D. S. Orientation of Ferroelectric Domains and Disappearance upon Heating Methylammonium Lead Triiodide Perovskite from Tetragonal to Cubic Phase. ACS Appl. Mater. Interfaces 2018, 1, 1534–1539. 10.1021/acsaem.7b00330.

[ref20] Bernasconi A.; Page K.; Dai Z.; Tan L. Z.; Rappe A. M.; Malavasi L. Ubiquitous Short-Range Distortion of Hybrid Perovskites and Hydrogen-Bonding Role: the MAPbCl_3_ Case. J. Phys. Chem. C 2018, 122, 28265–28272. 10.1021/acs.jpcc.8b10086.

[ref21] Drużbicki K.; Pinna R. S.; Rudić S.; Jura M.; Gorini G.; Fernandez-Alonso F. Unexpected Cation Dynamics in the Low-temperature Phase of Methylammonium Lead Iodide: the Need for Improved Models. J. Phys. Chem. Lett. 2016, 7, 4701–4709. 10.1021/acs.jpclett.6b01822. 27804302

[ref22] Drużbicki K.; Lavén R.; Armstrong J.; Malavasi L.; Fernandez-Alonso F.; Karlsson M. Cation Dynamics and Structural Stabilization in Formamidinium Lead Iodide Perovskites. J. Phys. Chem. Lett. 2021, 12, 3503–3508. 10.1021/acs.jpclett.1c00616. 33792334

[ref23] Drużbicki K.; Gaboardi M.; Fernandez-Alonso F. Dynamics & Spectroscopy with Neutrons—Recent Developments & Emerging Opportunities. Polymers 2021, 13, 1440–44. 10.3390/polym13091440. 33947108 PMC8125526

[ref24] Tkatchenko A.; Scheffler M. Accurate Molecular van der Waals Interactions from Ground-State Electron Density and Free-Atom Reference Data. Phys. Rev. Lett. 2009, 102, 073005–4. 10.1103/PhysRevLett.102.073005. 19257665

[ref25] Grimme S.; Ehrlich S.; Goerigk L. Effect of the Damping Function in Dispersion Corrected Density Functional Theory. J. Comput. Chem. 2011, 32, 1456–1465. 10.1002/jcc.21759. 21370243

[ref26] Becke A. D.; Johnson E. R. A Density-functional Model of the Dispersion Interaction. J. Chem. Phys. 2005, 123, 154101–9. 10.1063/1.2065267. 16252936

[ref27] Perdew J. P.; Ruzsinszky A.; Csonka G. I.; Vydrov O. A.; Scuseria G. E.; Constantin L. A.; Zhou X.; Burke K. Restoring the Density-Gradient Expansion for Exchange in Solids and Surfaces. Phys. Rev. Lett. 2008, 100, 136406–4. 10.1103/PhysRevLett.100.136406. 18517979

[ref28] Bartók A. P.; Yates J. R. Regularized SCAN functional. J. Chem. Phys. 2019, 150, 161101–5. 10.1063/1.5094646. 31042928

[ref29] Sun J.; Ruzsinszky A.; Perdew J. Strongly Constrained and Appropriately Normed Semilocal Density Functional. Phys. Rev. Lett. 2015, 115, 036402–6. 10.1103/PhysRevLett.115.036402. 26230809

[ref30] Bokdam M.; Lahnsteiner J.; Ramberger B.; Schäfer T.; Kresse G. Assessing Density Functionals Using Many Body Theory for Hybrid Perovskites. Phys. Rev. Lett. 2017, 119, 145501–5. 10.1103/PhysRevLett.119.145501. 29053325

[ref31] Lahnsteiner J.; Kresse G.; Heinen J.; Bokdam M. Finite-temperature Structure of the MAPbI_3_ perovskite: Comparing Density Functional Approximations and Force Fields to Experiment. Phys. Rev. Mater. 2018, 2, 073604–14. 10.1103/PhysRevMaterials.2.073604.

[ref32] Pérez-Osorio M. A.; Champagne A.; Zacharias M.; Rignanese G.-M.; Giustino F. Van der Waals Interactions and Anharmonicity in the Lattice Vibrations, Dielectric Constants, Effective Charges, and Infrared Spectra of the Organic-Inorganic Halide Perovskite CH_3_NH_3_PbI_3_. J. Phys. Chem. Lett. 2017, 121, 18459–18471. 10.1021/acs.jpcc.7b07121.

[ref33] Zhang D.; Hu X.; Chen T.; Abernathy D. L.; Kajimoto R.; Nakamura M.; Kofu M.; Foley B. J.; Yoon M.; Choi J. J.; et al. Temporally Decoherent and Spatially Coherent Vibrations in Metal Halide Perovskites. Phys. Rev. B 2020, 102, 224310–10. 10.1103/PhysRevB.102.224310.

[ref34] Fernandez-Alonso F., Price D. L., Eds.; Neutron Scattering - Fundamentals; Academic Press: New York, 2013.

[ref35] Fernandez-Alonso F.; Price D. L. Neutron Scattering - Applications in Biology, Chemistry, and Materials Science; Academic Press: New York, 2013.

[ref36] Manley M. E.; Hong K.; Yin P.; Chi S.; Cai Y.; Hua C.; Daemen L. L.; Hermann R. P.; Wang H.; May A. F.; Asta M.; Ahmadi M. Giant Isotope Effect on Phonon Dispersion and Thermal Conductivity in Methylammonium Lead Iodide. Sci. Adv. 2020, 6, eaaz1842–9. 10.1126/sciadv.aaz1842. 32789169 PMC7399528

[ref37] Moratalla M.; Gebbia J. F.; Ramos M. A.; Pardo L. C.; Mukhopadhyay S.; Rudić S.; Fernandez-Alonso F.; Bermejo F. J.; Tamarit J. L. Emergence of Glassy Features in Halomethane Crystals. Phys. Rev. B 2019, 99, 024301–9. 10.1103/PhysRevB.99.024301.

[ref38] Fabini D. H.; Hogan T.; Evans H. A.; Stoumpos C. C.; Kanatzidis M. G.; Seshadri R. Dielectric and Thermodynamic Signatures of Low-Temperature Glassy Dynamics in the Hybrid Perovskites CH_3_NH_3_PbI_3_ and HC(NH_3_)_3_PbI_3_. J. Phys. Chem. Lett. 2016, 7, 376–381. 10.1021/acs.jpclett.5b02821. 26763606

[ref39] Mozur E. M.; Maughan A. E.; Cheng Y.; Huq A.; Jalarvo N.; Daemen L. L.; Neilson J. R. Orientational Glass Formation in Substituted Hybrid Perovskites. Chem. Mater. 2017, 29, 10168–10177. 10.1021/acs.chemmater.7b04017.

[ref40] Simenas M.; Balciunas S.; Wilson J. N.; Svirskas S.; Kinka M.; Garbaras A.; Kalendra V.; Gagor A.; Szewczyk D.; Sieradzki A.; et al. Suppression of Phase Transitions and Glass Phase Signatures in Mixed Cation Halide Perovskites. Nat. Commun. 2020, 11, 5103–9. 10.1038/s41467-020-18938-z. 33037192 PMC7547736

[ref41] Kawachi S.; Atsumi M.; Saito N.; Ohashi N.; Murakami Y.; Yamaura J. Structural and Thermal Properties in Formamidinium and Cs-Mixed Lead Halides. J. Phys. Chem. Lett. 2019, 10, 6967–6972. 10.1021/acs.jpclett.9b02750. 31645099

[ref42] Clark S. J.; Segall M. D.; Pickard C. J.; Hasnip P. J.; Probert M. I. J.; Refson K.; Payne M. C. First Principles Methods Using CASTEP. Z. Kristallogr. 2005, 220, 567–570. 10.1524/zkri.220.5.567.65075.

[ref43] Lloyd-Williams J. H.; Monserrat B. Lattice Dynamics and Electron-phonon Coupling Calculations Using Nondiagonal Supercells. Phys. Rev. B 2015, 92, 184301–9. 10.1103/PhysRevB.92.184301.

