# Supplementary material for: Unraveling the Ordered Phase of the Quintessential Hybrid Perovskite MAPbI3—Thermophysics to the Rescue
Source: J Phys Chem Lett. 2022 Sep 7;13(36):8422–8. doi: 10.1021/acs.jpclett.2c02208 (PMC9486940; doi:10.1021/acs.jpclett.2c02208)
Supplement: Supplementary file 1 — jz2c02208_si_001.pdf [file jz2c02208_si_001.pdf]

Supplementary Information for Publication: Unravelling the Ordered Phase of the Quintessential Hybrid Perovskite $MAPbI_3$ – Thermophysics to the Rescue

Pelayo Marín Villa,[†] Ana Arauzo,[‡] Kacper Drużbicki,[*,†,¶] and Felix Fernandez-Alonso[*,†,§,‖]

†*Materials Physics Center, CSIC-UPV/EHU, Paseo Manuel de Lardizabal 5, 20018 Donostia - San Sebastian, Spain*

‡*Instituto de Nanociencia y Materiales de Aragón (INMA), CSIC-Universidad de Zaragoza, Pedro Cerbuna 12,50009 Zaragoza, Spain*

¶*Polish Academy of Sciences, Centre of Molecular and Macromolecular Studies, Sienkiewicza 112, 90-363 Lodz, Poland*

§*Donostia International Physics Center (DIPC), Paseo Manuel de Lardizabal 4, 20018 Donostia - San Sebastian, Spain*

‖*IKERBASQUE, Basque Foundation for Science, Plaza Euskadi 5, 48009 Bilbao, Spain*

E-mail: kacper.druzbicki@ehu.eus; felix.fernandez@ehu.eus

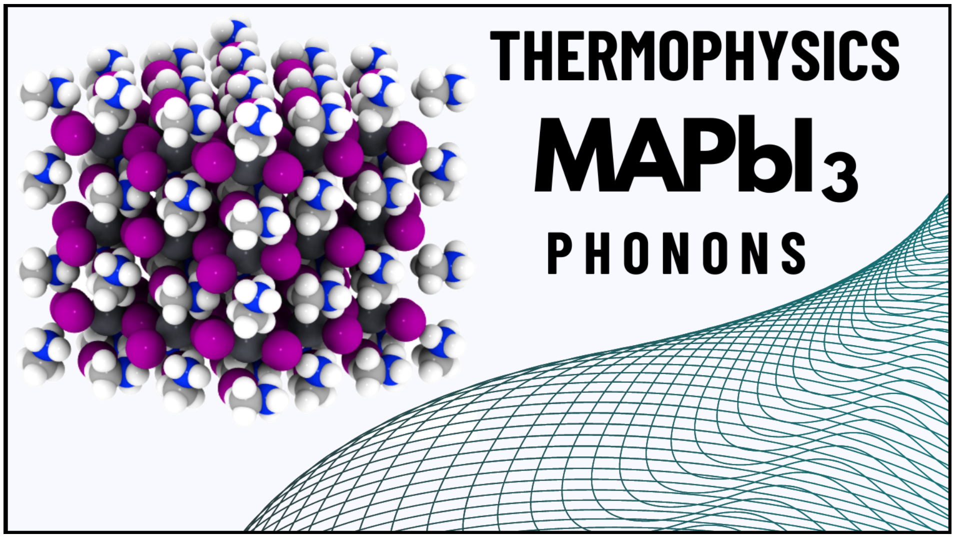

S1. Computational Details

The Plane-Wave Pseudo-Potential (PW-PP) formulation of Density Functional Theory (DFT) under Periodic Boundary Conditions (PBCs) was used along with the developer´s version of the CASTEP code.[1,2] We used three different semi-local Density Functional Theory Approximations (DFAs), namely, two exchange-correlation functionals within the Generalized-Gradient-Approximation (GGA), i.e. the original incarnation of the Perdew-Burke-Ernzerhof (PBE)[3] functional along with its solid-state version, PBEsol.[4] Furthermore, we employed the regularized version (rSCAN)[5] of the meta-GGA-type SCAN functional.[6] The core electrons were described by a set of hard norm-conserving PPs, while the electronic wave functions were defined using a PW basis set with a kinetic energy cutoff of 900 eV. The pseudopotentials were generated on-the-fly using the parent functional in all cases but in the rSCAN calculations, where PBE pseudopotentials were used instead. For the PBE functional we adopted two different atom-pair-wise approximations to account for van-der-Waals (vdW) interactions in our calculations: Tkatchenko-Scheffler corrections, DFT-vdW(TS);[7] and the D3 correction method of Grimme *et al.* with Becke-Johnson (BJ) damping, DFT-D3(BJ), the latter including three-body terms.[8–10]

The numerical settings were the same as those used in our previous studies on $MAPbI_3$.[11,12] A Monkhorst-Pack grid was used to maintain a constant k-spacing of 0.05 Å^{-1}. All structures were accurately relaxed at atmospheric pressure to minimize residual atomic forces. The convergence criteria in the variation of the total energy, Hellmann–Feynman forces, external stress, maximum displacement, and the self-consistent field (SCF) were defined as 1×10^{-12} eV/atom, 1×10^{-5} eV/Å, 0.0001 GPa, 1×10^{-6} Å, and 1×10^{-12} eV/atom, respectively. Following geometry optimization, phonon frequencies and eigenvectors were calculated from dynamical matrices constructed by numerical differentiation of the analytical gradients with respect to atomic displacements. For the finite-difference calculations, we used a displacement amplitude of 0.01 Å. The non-diagonal supercell method by Lloyd-Williams and Monserrat was employed to reduce the size of the supercell required to obtain the force constants.[13] The calculated phonon band structure gives us the harmonic energy $E_{\mathbf{k}j}$ of a given mode j for a given wave-vector $\mathbf{k}$. The Vibrational Density of States (VDoS) is then an explicit function of energy, defined as the number of modes per unit energy. Likewise, the heat capacity at constant volume is given by the following double-summation

$$C_V(T) = \sum_{\mathbf{k},j} \frac{exp(E_{\mathbf{k}j}/k_BT)}{\left[exp\left(E_{\mathbf{k}j}/k_BT\right) - 1\right]^2} \left(\frac{E_{\mathbf{k}j}}{k_BT}\right)^2, \tag{1}$$

where T is the temperature and k_B is Boltzmann´s constant. As written, C_V is an adimensional quantity that is equal to zero at T=0 and is bounded from above by the Dulong-Petit limit at high temperature. This summation can be restricted arbitrarily, in order to obtain partial contributions to the heat capacity over a given energy range, as illustrated in Figs. 2c and 2d in the main text.

The experimentally accessible specific-heat capacity is obtained at constant pressure, $C_p(T)$. $C_p(T)$ is related to $C_V(T)$ by the following equation:[14]

$$C_p(T) = C_V(T) + \alpha_V(T)^2\, B_0(T)\, V(T)\, T \tag{2}$$

where $B_0(T)$ is the bulk modulus, $V(T)$ is the volume at temperature T, and α_V is the volumetric thermal expansion coefficient. The second term proportional to temperature on the right-hand side of Equation (3) is the so-called lattice-dilatation term, which is negligible at the low-temperature conditions of relevance to the present work. Hence, $C_p(T) \approx C_V(T)$. To compare with computational predictions, the experimentally obtained molar $C_p(T)$ has been normalized by the universal gas constant R.

S2. Deviations and Statistical Methods

First, a spline interpolation was performed between the (denser) computational data sets obtained for each DFA. This operation allowed us to compare the experimental measurements and the computational predictions at the same temperatures. Consequently, the differences $\Delta C_p/T^3$ were obtained as:

$$\Delta C_p/T^3 = y_i - x_i \tag{3}$$

where y_i and x_i stand for the predicted and the experimental values, respectively.

Finally, the (cumulative) Mean Absolute Errors (MAE) in the prediction of the 'Debye-reduced' heat-capacity values were computed using:

$$\text{MAE} = \frac{1}{n}\sum_{i=1}^{n} |y_i - x_i| \tag{4}$$

where n is the size of the data set (in this case, the number of experimental points).

S3. Additional Computational Results

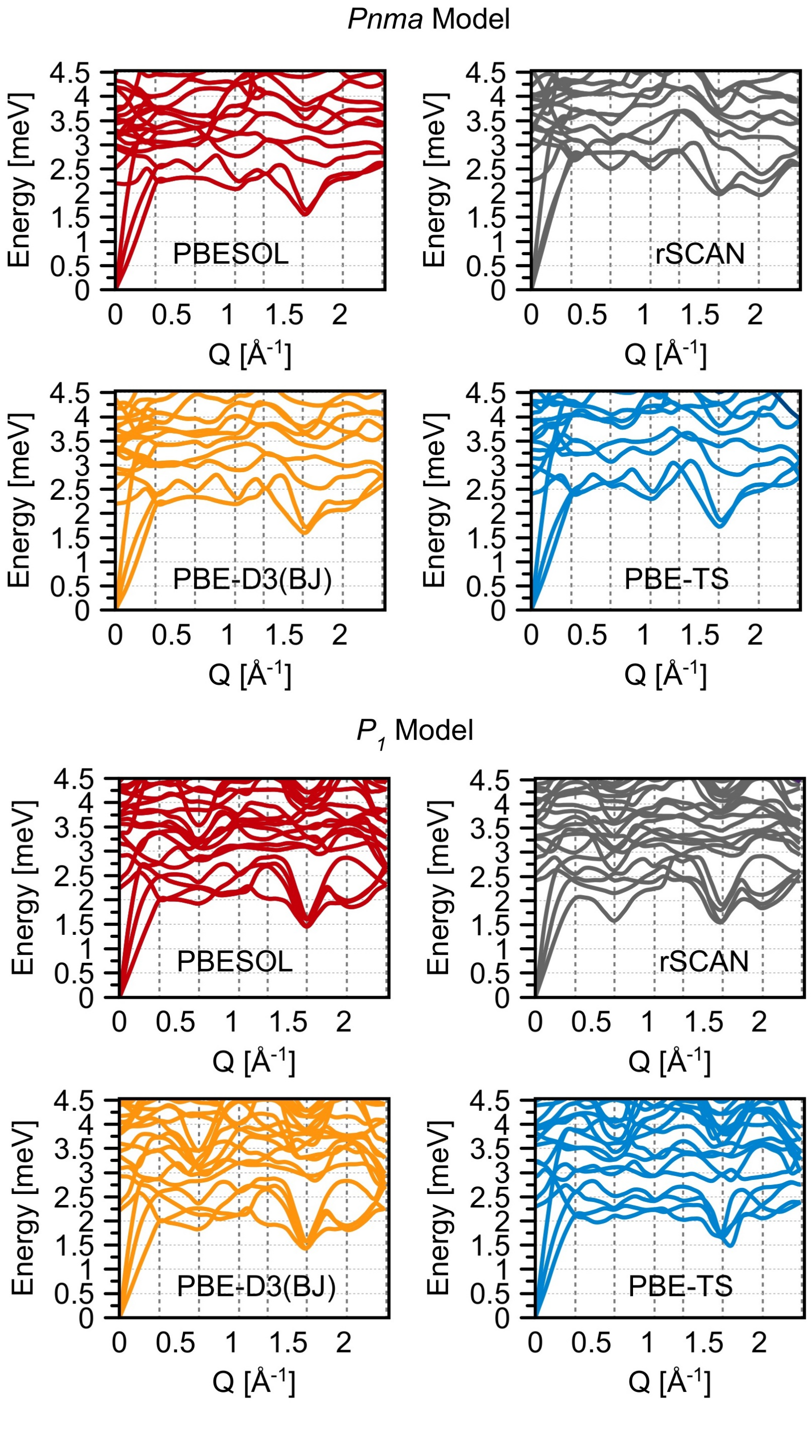

Figure S1: Phonon-dispersion relations calculated for the *Pnma* and *P*1 models of the ordered phase of $MAPbI_3$, using a range of DFAs.

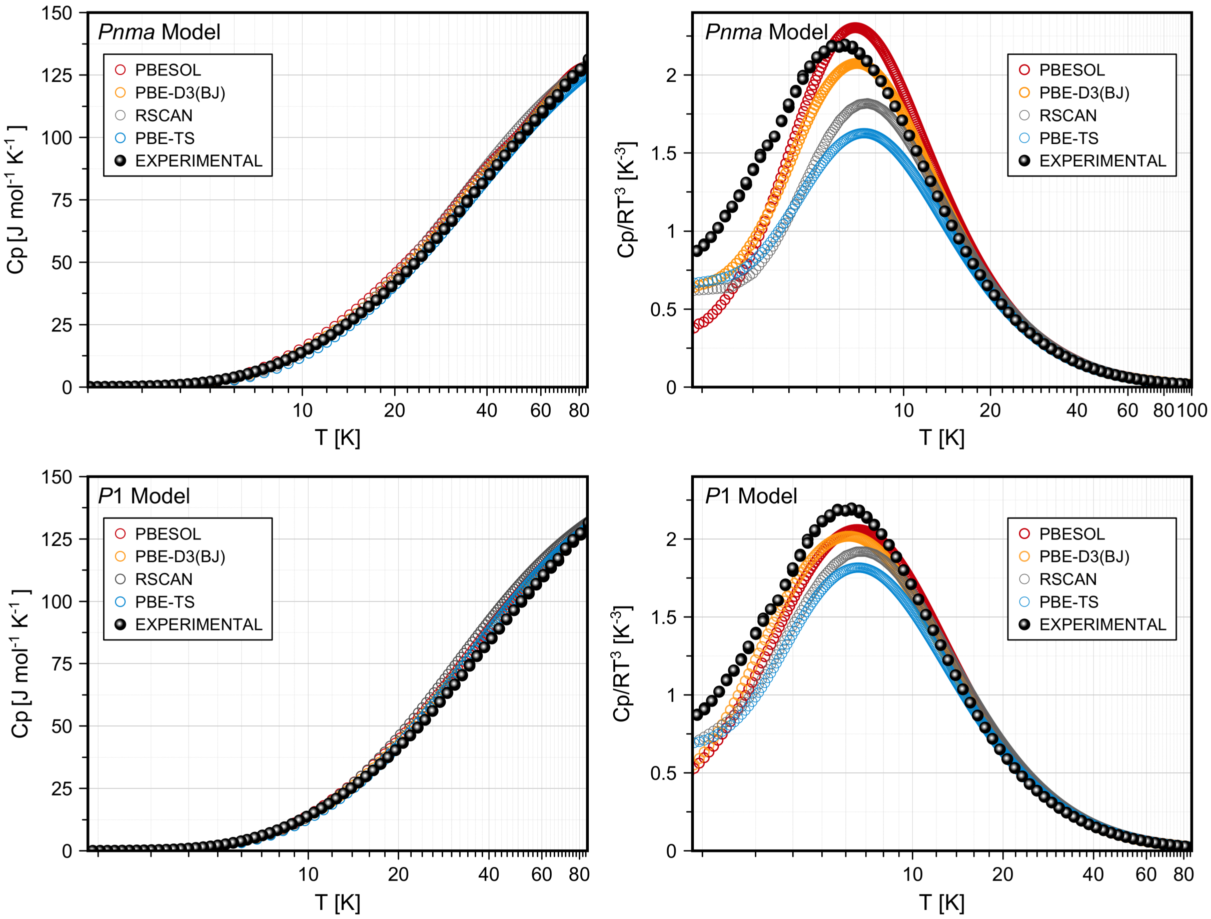

Figure S2: Experimental (black dots) and computational (colored empty circles) thermophysical data: C_p (left panels) and $C_p(T)/T^3$ (right panels). The calculations have been performed using the DFAs reported in Fig. S1.

References

(1) Payne, M. C.; Teter, M. P.; Allan, D. C.; Arias, T.; Joannopoulos, J. D. Iterative Minimization Techniques for Ab Initio Total-energy Calculations - Molecular-Dynamics and Conjugate Gradients. *Rev. Mod. Phys.* **1992**, *64*, 1045–1097.

(2) Clark, S. J.; Segall, M. D.; Pickard, C. J.; Hasnip, P. J.; Probert, M. I. J.; Refson, K.; Payne, M. C. First Principles Methods Using CASTEP. *Z. Kristallogr.* **2005**, *220*, 567–570.

(3) Perdew, J. P.; Burke, K.; Ernzerhof, M. Generalized Gradient Approximation Made

Simple. *Phys. Rev. Lett.* **1996**, *77*, 3865–4.

(4) Perdew, J. P.; Ruzsinszky, A.; Csonka, G. I.; Vydrov, O. A.; Scuseria, G. E.; Constantin, L. A.; Zhou, X.; Burke, K. Restoring the Density-Gradient Expansion for Exchange in Solids and Surfaces. *Phys. Rev. Lett.* **2008**, *100*, 136406–4.

(5) Bartók, A. P.; Yates, J. R. Regularized SCAN functional. *J. Chem. Phys.* **2019**, *150*, 161101–5.

(6) Sun, J.; Ruzsinszky, A.; Perdew, J. Strongly Constrained and Appropriately Normed Semilocal Density Functional. *Phys. Rev. Lett.* **2015**, *115*, 036402–6.

(7) Tkatchenko, A.; Scheffler, M. Accurate Molecular van der Waals Interactions from Ground-State Electron Density and Free-Atom Reference Data. *Phys. Rev. Lett.* **2009**, *102*, 073005–4.

(8) Grimme, S.; Antony, J.; Ehrlich, S.; Krieg, H. A Consistent and Accurate Ab Initio Parametrization of Density Functional Dispersion Correction (DFT-D) for the 94 Elements H-Pu. *J. Chem. Phys.* **2010**, *132*, 154104–19.

(9) Grimme, S.; Ehrlich, S.; Goerigk, L. Effect of the Damping Function in Dispersion Corrected Density Functional Theory. *J. Comput. Chem.* **2011**, *32*, 1456–1465.

(10) Becke, A. D.; Johnson, E. R. A Density-functional Model of the Dispersion Interaction. *J. Chem. Phys.* **2005**, *123*, 154101–9.

(11) Drużbicki, K.; Pinna, R. S.; Rudić, S.; Jura, M.; Gorini, G.; Fernandez-Alonso, F. Unexpected Cation Dynamics in the Low-Temperature Phase of Methylammonium Lead Iodide: The Need for Improved Models. *J. Phys. Chem. Lett.* **2016**, *7*, 4701–4709.

(12) Drużbicki, K.; Lavén, R.; Armstrong, J.; Malavasi, L.; Fernandez-Alonso, F.; Karlsson, M. Cation Dynamics and Structural Stabilization in Formamidinium Lead Iodide Perovskites. *J. Phys. Chem. Lett.* **2021**, *12*, 3503–3508.

(13) Lloyd-Williams, J. H.; Monserrat, B. Lattice Dynamics and Electron-phonon Coupling Calculations Using Nondiagonal Supercells. *Phys. Rev. B* **2015**, *92*, 184301–9.

(14) Iikubo, S.; Ohtani, H.; Hasebe, M. First-Principles Calculations of the Specific Heats of Cubic Carbides and Nitrides. *Mater. Trans.* **2010**, *51*, 574–577.
